# Supplementary material for: Use of Hair as Matrix for Trace Elements Biomonitoring in Cattle and Roe Deer Sharing Pastures in Northern Italy
Source: Animals (Basel). 2024 Jul 30;14(15):2209. doi: 10.3390/ani14152209 (PMC11311060; doi:10.3390/ani14152209)
Supplement: Supplementary file 1 [file animals-14-02209-s001.zip › animals-3081421-supplementary.pdf]

Supplementary informations

Table S1. Composition of the complentary feed administered to bovine. The feed was administered in the quantity of 2.5 kg/day. The ingredients quantity is expressed in kg.

Ingredient	Kg
Linseed	0.16
Roasted wholemeal soya	0.16
Hydrogenated lipids	0.1
Carbohydrates (Sugars)	0.2
NaBicarbonate + MgOxide	0.092
Minerals and Vitamins integration*	0.16
Saccharomyces cerevisiae	0.01
NDF	0.118
TOT	1

*ingredients of the supplement are reported in table S2.

Table S2. Composition of the supplement.

**Vitamins/pro-vitamins**	Unit	Value
Betacarotene 3s160(a)	mg	1.4
Vitamin A 3a672a	UI	560000
Vitamin D3 3a671	UI	21000
Vitamin E 3a700	mg	490
Vitamins B1 3a821	mg	2
Vitamin B2 3a831	mg	2.8
Vitamin B6 3a831	mg	1.4
Vitamin B12	mg	0.07
Calcium D-pantothenate 3a841	mg	3.5
Biotin 3a880	mg	6.3
Niacinamide 3a315	mg	28
Folic acid 3a316	mg	16.8
**Oligoelements Compounds**		
3b103 Iron sulphate [II] monohydrate - Fe	mg	14
3b203 Anhydrous calcium iodide in coated granules - I	mg	11.2
3b502 Manganese oxide [II] - Mn	mg	154
3b405 Copper [II] sulphate pentahydrate - Cu	mg	154
3b603 Zinc oxide - Zn	mg	420
3b605 Zinc sulphate monohydrate - Zn	mg	420
3b801 Sodium selenite - Se	mg	3.1

Table S3. Diet analytical composition.

Diet analytical data	% s.t.q.
Crude protein	15
Ether extract	20
Crude fiber	5
Ash	25
Na	3.12
Mg	1.19
